# Discrimination of Movement-Related Cortical Potentials Exploiting Unsupervised Learned Representations From ECoGs

**DOI:** 10.3389/fnins.2019.01248

**Published:** 2019-11-22

**Authors:** Carlos A. Loza, Chandan G. Reddy, Shailaja Akella, José C. Príncipe

**Affiliations:** ^1^Department of Mathematics, Universidad San Francisco de Quito, Quito, Ecuador; ^2^Instituto de Neurociencias, Universidad San Francisco de Quito, Quito, Ecuador; ^3^Department of Neurosurgery, University of Iowa, Iowa City, IA, United States; ^4^Department of Neurosurgery, University of Florida, Gainesville, FL, United States; ^5^Computational NeuroEngineering Lab, Electrical and Computer Engineering Department, University of Florida, Gainesville, FL, United States

**Keywords:** brain-computer interfaces, electrocoticogram (ECoG), generative model, minimum description length (MDL), representation learning, temporal marked point process

## Abstract

Brain–Computer Interfaces (BCI) aim to bypass the peripheral nervous system to link the brain to external devices via successful modeling of decoding mechanisms. BCI based on electrocorticogram or ECoG represent a viable compromise between clinical practicality, spatial resolution, and signal quality when it comes to extracellular electrical potentials from local neuronal assemblies. Classic analysis of ECoG traces usually falls under the umbrella of Time-Frequency decompositions with adaptations from Fourier analysis and wavelets as its most prominent variants. However, analyzing such high-dimensional, multivariate time series demands for specialized signal processing and neurophysiological principles. We propose a generative model for single-channel ECoGs that is able to fully characterize reoccurring rhythm–specific neuromodulations as weighted activations of prototypical templates over time. The set of timings, weights and indexes comprise a temporal marked point process (TMPP) that accesses a set of bases from vector spaces of different dimensions—a dictionary. The shallow nature of the model admits the equivalence between latent variables and representations. In this way, learning the model parameters is a case of unsupervised representation learning. We exploit principles of Minimum Description Length (MDL) encoding to effectively yield a data-driven framework where prototypical neuromodulations (not restricted to a particular duration) can be estimated alongside the timings and features of the TMPP. We validate the proposed methodology on discrimination of movement-related tasks utilizing 32-electrode grids implanted in the frontal cortex of six epileptic subjects. We show that the learned representations from the high-gamma band (85–145 Hz) are not only interpretable, but also discriminant in a lower dimensional space. The results also underscore the practicality of our algorithm, i.e., 2 main hyperparameters that can be readily set via neurophysiology, and emphasize the need of principled and interpretable representation learning in order to model encoding mechanisms in the brain.

## 1. Introduction

Brain-Computer Interfaces (BCI) strive to surpass the need for any measure of muscle control in order to provide patients suffering from severe neuromuscular disabilities with the ability to interact with the external world. These systems are anchored on principled analysis of the electrical activity of the brain during movement or movement intent; successful decoding of such neurophysiological processes is then relayed to external devices that execute the desired motor activity (Lebedev and Nicolelis, 2006). Recent technological and scientific advances in BCI systems have extended its application from enabling communication for completely “locked in” patients (Kübler et al., 2001; Vansteensel et al., 2016; Chaudhary et al., 2017), to restoration of motor control for patients with severe disabilities (Pfurtscheller et al., 2000; Hochberg et al., 2012; Yanagisawa et al., 2012; Ajiboye et al., 2017), and neurorehabilitation where BCIs are doubled as therapeutic devices (Dobkin, 2007; Soekadar et al., 2015; Bundy et al., 2017).

Current BCIs most commonly depend on scalp electroencephalogram (EEG) to record the combined electrical potentials of massive neuronal populations. While EEG is a non-invasive and cost-effective alternative, it is limited both in terms of spatial and temporal resolutions due to the overlapping activity of different cortical generators. In addition, the passive conductance through brain tissue, bone, and skin restrict the effective spectral support of the EEGs (Lebedev and Nicolelis, 2006). BCI systems depending on other non-invasive methods like magnetoencephalography (MEG) and functional magnetic resonance imaging (fMRI) provide finer spatiotemporal and spatial resolution, respectively (Weiskopf et al., 2004; Mellinger et al., 2007). However, besides being technically exhaustive, these methods are not cost effective. Moreover, the dependence of fMRI and positron emission tomography (PET) techniques on blood flow causes these systems to have very long time constants deeming them impractical for rapid communication and closed-loop applications (Vaughan, 2003).

Invasive methods involving single and multiunit recordings circumvent all the above mentioned drawbacks while delivering outstanding performance (Serruya et al., 2002; Taylor et al., 2002; Lebedev et al., 2005; Hochberg et al., 2012; Collinger et al., 2013; Bouton et al., 2016). However, these methods require that the cortex be penetrated which brings into question the safety of such technologies. Further, glial scars may develop overtime decreasing accessibility of units and inducing complex histological activity, simultaneously debilitating neural recordings. Finally, spatial resolution is inherently limited due to the restricted surface area covered by the recording electrodes (Abdulkader et al., 2015; Waldert, 2016).

Considering the disadvantages of both invasive and non-invasive BCIs and keeping in mind the ultimate aim of designing a durable, fully-implantable BCI system, many research groups have suggested Electrocorticogram (ECoG) as a more practical solution. These signals are acquired by implanting a grid of flat electrodes either above or below the dura mater, while never actually penetrating the brain parenchyma. Number of electrodes in these grids vary between 4 and 256, each having a diameter between 70 and 2 mm and an inter-electrode spacing between 1 and 10 mm depending on the extent of coverage and precision appropriate for analysis (Schalk and Leuthardt, 2011). Commonly used for invasive monitoring in patients with epilepsy (Reddy et al., 2009; Tangermann et al., 2012; Arya et al., 2017), these electrodes measure the cumulative activity of multiple neurons present in a small radius (~ 50–350 μm) around the tip of the electrode. Given their proximity to the brain surface, ECoG recordings not just provide better spatial resolution (1.2–1.4 mm compared to several cm in EEG), improved SNR and larger spectral support (0–500 vs. 0–40 Hz in EEG), they have also been found to be more robust to electrooculographic (EOG) and electromyographic (EMG) artifacts (Freeman et al., 2000; Ball et al., 2009). Moreover, while fidelity and durability of these electrodes have been positively tested in macaques for several months (Chao et al., 2010; Mestais et al., 2015; Degenhart et al., 2016), further evaluation on a group of patients implanted with subdural electrodes is under experimentation (Delavallée et al., 2008). ECoG recordings, therefore, strike a perfect balance between clinical practicality and signal quality, consequently delivering prominence in performance (Leuthardt et al., 2006; Schalk et al., 2008; Kubanek et al., 2009; Brunner et al., 2011; Yanagisawa et al., 2012; Hotson et al., 2016; Degenhart et al., 2018).

The broad spectral support available via ECoG recordings has important implications for BCI applications pertaining to encoding and decoding motor tasks. For instance, increased modulatory activity of faster rhythms (75–100 Hz) in the motor cortex of patients performing sustained muscle contractions has shown specific somatotopic organization (Crone et al., 1998; Miller et al., 2007). Several ECoG-based studies have confirmed the correlation between spatially focused gamma activity and motor function (Aoki et al., 1999; Miller et al., 2010; Leuthardt et al., 2012; Gunduz et al., 2016). Although advances in recording technology has allowed for similar EEG-based (Jokeit and Makeig, 1994; Darvas et al., 2010), the recordings usually suffer from severe contamination due to muscle artifacts (Goncharova et al., 2003).

In addition to the BCI recording paradigm, appropriate signal processing and feature extraction are paramount for designing effective BCIs. Extracellular electrical potentials from the brain—such as EEG, ECoG, and Local Field Potentials (LFP)—are usually deemed as either chaotic deterministic or stochastic non–stationary sequences; hence, they require principled and distinct processing that needs to incorporate neurophysiological principles into the modeling framework. Neuromodulations, also known as phasic events, wave packets, or micro-events constitute an order parameter of neuronal assemblies in the sense that the population imposes order by regulated synaptic interactions, i.e., they reflect the spatiotemporal interplay of local neuronal populations (Freeman and Quiroga, 2012). These textured images (as coined by Walter J. Freeman) appear in the recorded trace as organized, transient patterns and differ statistically from the featureless noisy background known to be characterized by a 1/*f* power spectrum (Freeman, 1975). Moreover, phasic events and deviation of Normality are the telltale signs of self-organized criticality—a metastable state of the brain that allows shifting between dynamical states (Buzsaki, 2006). The goal of signal processing is, then, to discriminate between relevant neuromodulations and the temporally disorganized but spatially structured background activity in order to elucidate the encoding mechanisms that arise during BCI tasks.

A vast majority of ECoG-based BCIs exploit Time-Frequency (TF) decompositions to build multiscale representations (Dat et al., 2006; Zhao et al., 2010; Aydemir and Kayikcioglu, 2011; Herff et al., 2016), while the more advanced population algorithms incorporate spatial information to account for propagation and dependencies across electrodes (Ince et al., 2008; Chao et al., 2010; Onaran et al., 2011; Ramsey et al., 2018). However, TF methods are limited in their performance pertaining to the uncertainty principle (Gabor, 1946) which lower bounds the product of time and frequency resolutions. That is, in order to efficiently capture short locally stationary segments from non-stationary ECoGs, one must utilize small evolving windows, which, then, compromises the frequency resolution of the representation and blurs the phase information of potential phasic events inside the processing window in question. Although, wavelets attempt to alleviate this shortcoming (Unser and Aldroubi, 1996; Mallat, 1999), the output still suffers due to the imposition of fixed structures on the analysis of the input signal, i.e., the inference is generic by nature due to the templates of the underlying imposed generative model. Lastly, the background activity (which is sometimes deemed as “noise” by the signal processing algorithms applied to each lead) demands for application-specific frameworks that explicitly model the physiological regimes embedded in the temporal traces. The resolution constraints of TF methods and the inference on generic generative models that disregard the complex dynamics of ECoG (e.g., linear projections onto preset sinusoids in the case of Fourier analysis) are the two main deterrents of TF decompositions. It is imperative to exploit the neurophysiology behind ECoG in order to propose principled generative models that would not only advance signal processing applied to Neuroengineering, but also exploit the multivariate nature of the ensembles in order to improve performance and interpretability of ECoG-based BCIs.

We exploit a data-driven framework based on a generative model for single-channel ECoGs which is able to fully characterize each scale-specific neuromodulation by its timing, amplitude, and duration (Loza et al., 2017). One of the main advantages of the generative model is its exceptional temporal resolution limited only by the sampling rate, i.e., no windowing is necessary. Inference on the model can be viewed as either classic feature engineering or sampling of a Temporal Marked Point Process (TMPP) (Daley and Vere-Jones, 2007) fully characterized by the intensity function of the timings and the joint probability density function (pdf) of the amplitudes and durations—the “features” of the TMPP. This dual interpretation opens the door to uncover novel encoding mechanisms beyond the pervasive power-modulation-based techniques. Learning on the model invokes neurophysiological principles to restrict the search space of potential phasic events by isolating the pervasive background component of extracellular electrical potentials (Freeman and Quiroga, 2012). Then, the resulting vector space is partitioned in a top-down approach by means of a greedy clustering scheme based on the principle of Minimum Description Length (MDL) (Grünwald, 2007). The outcome is a set of prototypical vectors from different vector spaces (i.e., durations)—a collection of cluster centroids that represent bona fide transient events. Lastly, the learning process is virtually parameter free: it only requires two main hyperparameters; however, they are tightly connected to the oscillatory rhythm under consideration and, thus, can be selected based on empirical rules fully supported by clinical and research fields.

The present study integrates the advantages of an ECoG-based BCI and the proposed unsupervised learning framework to discriminate movement-related tasks in six patients. Each subject was requested to perform a motor task involving moving a joystick in one of four directions (up, down, left, or right) and an additional finger movement “trigger” task while ECoG activity from two main areas are recorded. Labeled single-channel, multi-trial ensembles go through the learning and inference processes on the generative model with a focus on the high-gamma band (85–145 Hz). The results in terms of movement direction separability not only confirm the plausibility of the methods, but they also reveal a novel cortical encoding mechanism taking place during movement-related tasks. The rest of the paper continues as follows: section 2 explains the generative model for ECoG alongside the proposed learning mechanisms. Section 3 details the experimental setting, while section 4 summarizes the main results. Section 5 offers discussion, limitations, and perspective. Lastly, section 6 concludes the paper and proposes future work.

## 2. Experimental Setting

The study comprised of 3 male and 3 female participants in the age range of 22–40 years. All six subjects, suffering from medically intractable epilepsy, were undergoing invasive subdural electrode monitoring before resection. A standard (1 cm interelectrode spacing) 32-contact frontal grid and a high-density (0.5 cm interelectrode spacing) 96-contact temporal grid were used to ensure unilateral, frontotemporal, subdural grid coverage on the side corresponding to suspected seizure onset. Altogether, there were three patients with left coverage while the rest had right coverage. Patients did not incur additional risk by participating in these studies. Research protocols were approved by the University of Iowa Human Subjects Review Board.

**Figure 1:**
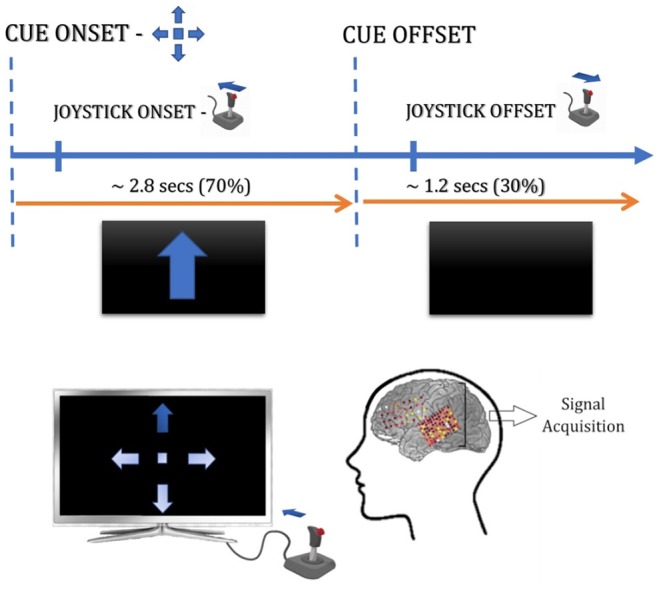
Experimental paradigm of ECoG–based BCI.

During the trials, each participant was instructed to move a joystick in one of the four cardinal directions (up, down, left, right) in response to a visual display of an arrow pointing toward the target location. A fifth display in the form of a square was also included as a “trigger condition” where in response to the cue, the participant was required to click the trigger button on the joystick with the tip of the index finger. All cues were randomly interleaved and no bias was introduced during their presentation. Further, the patient was required to hold the joystick in the target location until the visual display was replaced with a blank screen, following which the patient was asked to either release the joystick or bring it to a neutral position (Figure 1).

All trials lasted ~4 s. where the initial ~2.8 s involved stimulus presentation and joystick maneuvers, while in the remaining ~ 1.2 s the patient returned the joystick to its neutral position. All participants performed an average of 50 trials for each direction and “trigger” condition and all the trials were performed with the hand contralateral to the grid placement. Table S1 details the number of trials for each joystick direction under consideration in our study. All signals were acquired at a sampling rate of 2034.5 Hz, which were later downsampled to 500 Hz for analysis.

## 3. Methods

### 3.1. Generative Model for ECoG

Observable ECoG traces are the result of an underlying multiscale system that describes large-scale function of neuronal populations. One of the consequences of the structural fractal nature of the cortex is reflected on the very own fractal, scale-free nature of its observable variables (Buzsaki, 2006), being ECoG—with its characteristic 1/*f* law—one of the most representatives at a mesoscopic level. Self organized-criticality (Linkenkaer-Hansen et al., 2001; Freeman et al., 2003; Stam and De Bruin, 2004; Bak, 2013) further formalizes these concepts posing that brain dynamics remain at a complex state at the border between unpredictable chaos and predictable periodic behavior. The former representing a hypersensitive metastable state of the network near phase transitions, whereas the latter brings organization and transient stability by oscillations (Buzsaki, 2006). This type of micro-events have been well documented in the literature under the umbrella of induced potentials or event-related oscillations (Tallon-Baudry and Bertrand, 1999; Freeman and Quiroga, 2012), e.g., the occipital alpha rhythm (Berger, 1929), K-complexes, sleep spindles (Rechtschaffen et al., 1968), gamma oscillations in the olfactory bulb of cats and rabbits (Freeman, 1975), high-frequency oscillations correlated to the binding of perceptual information (Rodriguez et al., 1999), and hippocampal sharp-wave ripples (Buzsáki, 2015) to name a few. There are also so-called pathological patterns that are associated to particular states in a pathological setting, e.g., in epilepsy, inter-ictal spikes and high-frequency oscillations (HFO) or ripples have been deemed as biomarkers and even potential predictors of seizures (Worrell et al., 2004; Staley et al., 2011; Jacobs et al., 2012). The challenge of principled signal analysis lies on the detection, modeling, and further unveiling of the behavioral correlates of said events.

Walter J. Freeman posited that the physiological regimes of the generating local neural assembly are reflected on the statistical properties of its observable EEG traces (Freeman and Quiroga, 2012). If the network is at rest, the resulting EEG is featureless, unorganized, and with amplitudes that closely resemble a Gaussian distribution—a critical state characterized by expectation in the form of hypersensitivity to perturbations, such as sensory stimuli or motor output. Transition to an active or work state shifts the network dynamics, which is revealed by transient stability, and, in turn, derives in deviation form Gaussianity (according to higher–order statistical moments). The generating mechanisms behind extracellular electrical potentials guarantees seamless translation of Freeman's theories from EEG to more local (and invasive) electrophysiology, such as ECoG and LFP (Niedermeyer and da Silva, 2005; Buzsáki et al., 2012). Let ỹ(*t*) be the result of linear filtering a single-channel, single-trial ECoG trace. Linear filtering is necessary so that the Gaussian/Non-Gaussian regimes are preserved through linear operators on the raw signal. According to Freeman's experimental results and the theory of self-organized criticality of neuronal assemblies, ỹ(*t*) can be decomposed into two sequences:

(1) $$\begin{array}{l} \tilde{y}(t)=\{\begin{array}{ll} y(t) & \text{if Network is Active (Y State)}\\ z(t) & \text{if Network is at Rest (Z State)} \end{array} \end{array}$$

where *y*(*t*) is the phasic event component—an ideal, noiseless sequence that includes scale-specific neuromodulations over time. On the other hand, *z*(*t*) is the filtered version of the underlying ongoing activity, i.e., a background component.

The background component, *z*(*t*), ongoing or spontaneous activity is associated to rest regimes of the generating neural network. From a signal processing point of view, it can be regarded as noise due to its featureless nature. However, it should not be confused with interfering and external sources usually mixed and superimposed in ECoG recordings—the so-called artifacts, e.g., ocular and muscle activity, movement-related activity, signal degradation as a byproduct of variable electrode impedance, and so on (Niedermeyer and da Silva, 2005). Also, noise might imply a complete divorce from behavior, yet, several studies have confirmed the encoding nature of the ongoing EEG by regulating response variability and imposing priors for induced potentials (Başar, 1980; Buzsaki, 2006; Hanslmayr et al., 2006; Busch et al., 2009; Luczak et al., 2009). Moreover, the background component is essential to maintain cortical functions in a linear dynamic range (Freeman and Quiroga, 2012).

**Figure 2:**
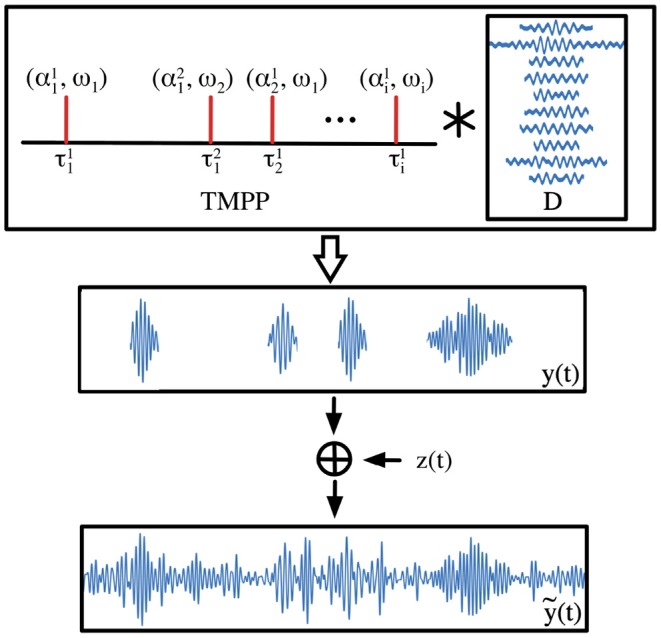
Generative model for ECoG. A bandpassed, single–channel, single–trial ECoG trace, ỹ(*t*), is modeled as the noisy addition of weighted, scale–specific, shifted filters over time. Temporal Marked Point Process (TMPP) samples sparsely activate dictionary atoms to yield the phasic event component *y*(*t*). Atom corresponding to ω_1_ appears twice in the TMPP. * represents indexed convolution according to Equation (2).

The phasic event component is modeled taking inspiration from the shot noise model (Davenport and Root, 1958). *y*(*t*) is the result of a Temporal Marked Point Process (TMPP) with timings τ and marks (features) α and ω activating filters, **d**, over time:

(2) $$\begin{array}{l} y\left(t\right)=\overset{K}{\underset{\omega =1}{\sum }}\overset{n_{\omega }}{\underset{i=1}{\sum }}\alpha_{i}^{\omega }{\text{d}}_{\omega }\left(t-\tau_{i}^{\omega }\right)+\epsilon \left(t\right) \end{array}$$

where $\text{D}={\left\{{\text{d}}_{\omega }\right\}}_{\omega =1}^{K}$ is a set of filters, kernels or atoms known as dictionary. $\tau_{i}^{\omega }$ and $\alpha_{i}^{\omega }$ are the timing and encoding coefficient of the *i*–th instance of filter **d**_ω_, respectively. ϵ(*t*) is the additive noise sequence (possibly resulting from thermal noise, variation in electrode impedance, and propagation losses through tissue). *n*_ω_ indicates the number of instances of **d**_ω_, which is not restricted to be the same across filters. ω basically constitutes an assigning set (i.e., index) between observed micro–events and modeled dictionary atoms, i.e., ω ∈ {1, 2, 3, …, *K*}. The resolution of τ is limited only by the recording sampling rate; for instance for 500 Hz, one can determine the occurrence of a neuromodulation with a 2 ms. resolution. In theory, the support of α is R; however, practical constraints are imposed by the power of the rhythm under consideration. Figure 2 illustrates the encoding from TMPP samples to noisy single-channel, bandpassed ECoG trace.

The model in (2) can be alternatively interpreted as *y*(*t*) being the observable variable from a generative model with latent variables *Y* and *Z*. *Y* is parameterized as Θ_*Y*_ ≜ {τ, α, ω, **D**}, whereas *Z*, being Gaussian in nature, is fully characterized by the mean and standard deviation of the background EEG, i.e. Θ_*Z*_ ≜ {μ_*Z*_, σ_*Z*_}. A multiple input single output (MISO) framework (Brockmeier and Príncipe, 2016) is the basis of the current generative model for ECoG; however, training was not scalable due to the amount of free hyperparameters. Then, a single-rhythm approach was adopted by Loza et al. (2017), where learning focused on scale-specific patterns of fixed duration by means of shift-invariant time series clustering techniques. The current work goes one step further and learns kernels of different lengths. Similar models have been proposed in neuroscience and statistics under the connotation of convolutional sparse coding (Lewicki, 2002; Smith and Lewicki, 2006; Balcan and Lewicki, 2009; Ekanadham et al., 2011). Their results highlight the need of principled priors and constraints to tackle an inherent combinatorial problem.

Given an ensemble of single-channel ECoG recordings, ${\left\{{ỹ}_{i}\left(t\right)\right\}}_{i=1}^{N}$. Learning on the model implies estimating the dictionary **D** whose elements, in general, are not restricted in duration—they represent bases from vector spaces of different dimensions. On the other hand, inference or encoding is posed as learning the set of timings and marks of the TMPP, i.e., sampling from a point process. The shallow nature of the model admits the equivalence between latent variables and features or representations. **D** also encodes features of its own, such as duration, central frequency, and Q-factor. Estimating Θ_*Y*_, then, can be posed as a case of unsupervised representation learning for ECoG (Bengio et al., 2013). The shallow generative framework and physiological-based constraints of the model guarantee that the learned dictionary and densities of timings, marks, and representations lead to meaningful and interpretable encoding mechanisms of the network without imposing handpicked signatures, as in the case of wavelets or Gabor bases.

### 3.2. Learning on the Model

**Figure 3:**
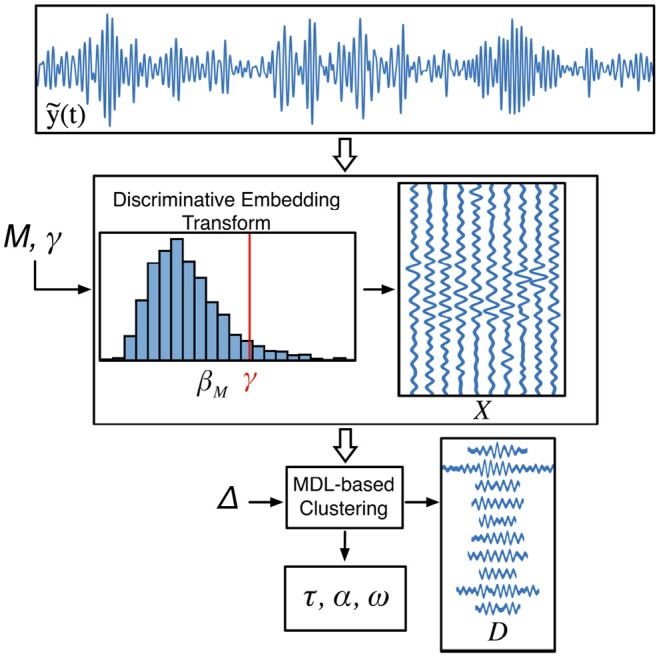
Learning of generating dictionary and TMPP features. The discriminative embedding transform (DET) isolates potential *M*–snippets generated by the *Y* (active) state and collects them in the matrix **X**. MDL–based hierarchical clustering estimates TMPP timings and marks as well as bases from vector spaces of different dimensions, **D**.

Estimating the latent variables of this type of generative models usually falls into two categories depending whether the sources are explicitly estimated or not during learning. Bell and Sejnowski (1996), Davies and James (2007), Lucena et al. (2011), and Brockmeier and Príncipe (2016) showcase the potential of learning the bases without appealing to reconstruction cost functions or explicitly estimating the sources, i.e., marks of the TMPP, by using Independent Component Analysis off–the–shelf implementations, such as FastICA (Hyvarinen, 1999). The alternative approach (adopted here) is to exploit block coordinate descent optimization to iteratively estimate the sources while keeping the filters fixed, and then, learn the dictionary atoms while keeping {τ, α, ω} fixed. The result is a local optimum in solution space with the added bonus of less computational demands. For a comparison of both approaches applied to a MISO model on synthetic and real EEG, refer to Brockmeier and Príncipe (2016). Achieving the global optimum is impossible in practice because it would require combinatorial analysis, which is simply intractable, i.e., it would require checking all the possible different combinations of dictionary atoms (with different dimensionalities) until optima are found; hence, here we opt for the tractable, albeit suboptimal solution to the problem at hand. For our case, learning takes place in two very distinctive sequential stages: discrimination between dynamical regimes and hierarchical partitioning of the data (Figure 3).

#### 3.2.1. Discrimination of Dynamical Regimes: From Traces to M–Snippets

We take advantage of the architectural constraints and neurophysiology of the ECoG to render the learning more tractable, alleviate the computational complexity, and, most importantly, facilitate the interpretation of the learned prototypes. This is accomplished by bandpass filtering the traces according to the clinical EEG rhythms (Niedermeyer and da Silva, 2005). The result is a natural grouping of scale-specific neuromodulations. Then, sparsity is leveraged by associating a single dictionary atom to an observed, noisy micro-event. This will further prevent overfitting and overlapped occurrences of TMPP samples; it also emphasizes the temporal sparsity of the sources. Then, there is major deviation from the approaches adopted in classic convolutional sparse coding applied to time series (Lewicki, 2002; Smith and Lewicki, 2006; Mailhé et al., 2008; La Tour et al., 2018). Instead of performing iterative template matching over time, e.g., Matching Pursuit (Mallat and Zhang, 1993), that attempts to reconstruct the entire input, we exploit Freeman's theories and the concept of self-organized criticality to map the ECoG to a surrogate space of constrained ℓ_2_-norms where discrimination between rest and active stages is plausible. Let the *M*-sample-long subsequence from ỹ[*n*] centered at the time instance *t* = *i* be a *M*-snippet:

(3) $$\begin{array}{l} \;{\tilde{y}}_{i}=\tilde{y}(i-M/2:i+M/2)\\ \text{s}.\text{t }i=M/2,M/2+1,...,\eta -M/2 \end{array}$$

where η is the number of sampled values in ỹ(*t*). One of the intermediate goals of learning on the generative model is to effectively discriminate between *M*-snippets generated by *Z* (background subsequences) and *M*-snippets with embedded phasic events generated by *Y*. The advantages of principled discrimination is two-fold: decrease likelihood of biased atoms and improved convergence rates by effective restriction of the input space to subsequences from the active stage, i.e., the learning is not reconstructive in nature because the entire ECoG trace, ỹ(*t*), is not worth encoding. The learning falls more into the hierarchical partitioning category. In this regard, the embedding transform (Loza and Principe, 2018)—introduced as a novel tool to assess non–stationarity of single-channel EEG ensembles—maps the input according to the ℓ_2_-norms of its constituent *M*-snippets. The *M*-snippets are built sequentially: first, modulated patterns are extracted (peak detection via moving averages or instantaneous amplitudes), then, the rest of the unmodulated patters complete the set of *M*-snippets. The middle points from each sample is collected in the set Π = {π_*i*_}. Powers of the *M*-dimensional vectors are calculated, and, the resulting ℓ_2_-norms comprise the surrogate variable β_*M*_. If ỹ(*t*) is strictly generated by *Z*, its amplitudes will resemble a Gaussian density, which derives in β_*M*_ being a chi-distribution with *M* degrees of freedom. Invoking the Central Limit Theorem, if *M* is large enough (which is satisfied for high sampling rates), the chi-distribution resolves to a Gaussian density. Conversely, *M*-snippets from *Y* interspersed between *Z* counterparts will drive the shape of β_*M*_ by enlarging the tails and skewing the distribution. β_*M*_ is then a surrogate variable of the dynamic stages of the network. The discriminative embedding transform (DET) goes one step further and proposes a threshold in the β_*M*_ space between potential *M*-snippets from different regimes. Specifically, the matrix **X** with columns **x**_*i*_ collects all the *M*-snippets larger than the hyperparameter γ:

(4) $$\begin{array}{l} \begin{array}{l} \;x_{i}=\tilde{y}{\left(\pi_{i}-M/2:\pi_{i}+M/2\right)}^{T}\\ \text{s}.\text{t}.\;||\tilde{y}(\pi_{i}-M/2:\pi_{i}+M/2){||}_{2}\geq \gamma \end{array} \end{array}$$

The case for a set of multi-trial recordings is straightforward, i.e., $X\in {\text{R}}^{M\times \Psi }$ where Ψ is the total number of *M*-snippets from ${\left\{{ỹ}_{i}\left(t\right)\right\}}_{i=1}^{N}$ generated by *Y*. In short, **X** collects potential embedded *M*-sample-long micro-events of single-channel, multi-trial traces according to a non-linear mapper with hyperparameters *M* and γ.

#### 3.2.2. Learning Bases of Different Dimensions

After **X** is computed, the naive solution to extract centers of mass in R^*M*^ would involve classic static clustering algorithms, e.g., k-means. Yet, the shift-invariance of embedded phasic events would most likely derive in meaningless clusters as noted in Keogh and Lin (2005). Most importantly, if prototypes of different durations are present, k-means would blur their contributions by grouping them in *M*-dimensional vectors. The former problem is addressed by cross-correlation operators: distances between prospective cluster centers and samples from **X** can now be estimated over lags (similar to Matching Pursuit implementations on time series). The latter problem is far more challenging; it demands for principled measures between centers, and vectors in general, of different dimensions, which is clearly prohibitive under Euclidean distance regimes. We exploit the parsimony principles of Minimum Description Length (MDL) coding to build a hierarchical partitioning in R^*M*^ where the learned atoms are not restricted to a fixed dimensionality.

The MDL principle is invoked to cluster reoccurring patterns embedded in the columns of **X**. The goal is to attempt to compress the data in a lossless manner by finding repeated structures (or regularities) in it. Due to inherent noise and response variability, we practically aim to discover repeated structure and encode the differences. For instance, the conditional description length of a sequence *A* after being encoded with a hypothesis *H* is given by *DL*(*A*|*H*) = *DL*(*A* − *H*); this can be interpreted as the cost of the encoding. *DL*(*T*) is the bit level representation of time series *T*, which is defined as the entropy of *T* times its length *m*, i.e.,:

(5) $$\begin{array}{l} DL\left(T\right)=-m\underset{t}{\sum }P\left(T=t\right)\log_{2}P\left(T=t\right) \end{array}$$

We exploit a cost function based on bit level representations to decide among three basic clustering operations: creating a cluster, adding a subsequence to an existing cluster, and merging clusters. This approach was first introduced in Rakthanmanon et al. (2011) under the term *time series epenthesis* as a virtually parameter-free framework to find repeated subsequences in time series without necessarily explaining all the data. In a similar manner, we sequentially build a hierarchical partition of the patterns embedded in **X** by greedily selecting the clustering operation that reduces the total number of bits saved after being applied, i.e., the difference in the number of bits before and after applying an operator—a *bitsave* (*BS*) cost function. At each iteration, one operator is selected and the updated set goes through the same process until the set **X** is exhausted. The BS corresponding to the three clustering operators are:

*BS* after creating cluster *C* from subsequences *A* and *B*:

(6) $$\begin{array}{l} BS=DL\left(A\right)+DL\left(B\right)-DLC\left(C\right) \end{array}$$

where $DLC(C)=DL(H)+\underset{A\in C}{\sum }DL(A|H)-\underset{A\in C}{\max}DL(A|H)$ is the number of bits needed to represent all subsequences assigned to cluster *C*. *H* is the center subsequence of the cluster under consideration.

*BS* after adding subsequence *A* to cluster *C*:

(7) $$\begin{array}{l} BS=DL\left(A\right)+DLC\left(C\right)-DLC\left(C^{\prime }\right) \end{array}$$

where *C*′ is the new cluster after adding *A* to *C*.

*BS* after merging clusters *C*_1_ and *C*_2_ into new cluster *C*′:

(8) $$\begin{array}{l} BS=DLC\left(C_{1}\right)+DLC\left(C_{2}\right)-DLC\left(C^{\prime }\right) \end{array}$$

Euclidean distance is used to initialize prospective clusters and find the closest subsequence from a given cluster center. Cross-correlation provides an intuitive extension to Euclidean distance over lags for both tasks and is, therefore, exploited in the current work. Consequently, the shift-invariance nature of the micro-events is explicitly modeled. Additional practical implementation details include quantizing the normalized inputs to a 64-bit representation so that *DL*s from different clusters and dimensions can be effectively compared. Also, the algorithm requires priors in the form of a set of prospective durations, δ ∈ Δ, in order to initialize cluster centers and initiate the optimization. Nevertheless, these priors are not rigid because cross-correlation operators are flexible enough to discover patterns beyond the grid imposed by Δ. Learning begins by finding the set of pairs mostly correlated in **X**—a sort of motif finding routine—for each δ. Querying these sets can be effectively executed by building matrices of sizes R^δ×δ^ with maximal pairwise cross–correlation as their elements. Initial cluster centers are merely the average between these motifs. Then, the operators of adding subsequences to existing clusters, merging clusters, and adding an existing motif to the active set are evaluated at each iteration. In summary, Δ are mere suggestions of dimensions to be explored initially, but, later during learning, the algorithm is free to venture into different dimensions up until the practical limit imposed by *M*. The timings, τ, are estimated as the lags corresponding to maximum cross–correlations with respect to the time stamp of **x**_*i*_ in the original time series. The encoding amplitudes or weights, α, are simply the aforementioned maximum cross-correlation values.

The proposed algorithm alternatively estimates the TMPP marks and learns bases from vector spaces of different dimensions. In this way, it resembles greedy block coordinate descent approaches. However, it is conceptually different from previous attempts to learn generating dictionaries for time series. First, it resembles the work in Rakthanmanon et al. (2011), in that we exploit MDL for hierarchical partitioning; yet, our implementation is significantly faster due to the pre-processing and discrimination of dynamical regimes provided by the DET. Second, clustering of shift-invariant patterns usually either fixes the support of possible discoverable patterns (Mailhé et al., 2008), or adapt this parameter in a semi-supervised manner (Lewicki, 2002; Smith and Lewicki, 2006; Loza et al., 2017). We propose a flexible initial grid that is free to be explored and shaped during learning as long as the bitsave is minimized. Lastly, and more importantly, the proposed clustering technique greedily selects the number of clusters, *K*, needed, i.e., model selection is a natural byproduct. This is a major improvement over classic convolutional sparse coders where *K* is left as a hyperparameter. The final implementation takes three main hyperparameters: γ, the threshold of dynamical regimes in the β_*M*_ space, *M*, the embedding dimension of the DET, and Δ, the duration prior. However, the last two are strictly tied to the rhythm under consideration; they can both be set according to previous studies, analysis of TF decompositions, or neurophysiology. γ is parameterized by the mean and standard deviation of the fitted Gaussian corresponding to *Z* in the β_*M*_ space, i.e., $\gamma =\mu_{Z_{M}}+\gamma^{\prime }\times \sigma_{Z_{M}}$ where μ_*Z*_*M*__ and σ_*Z*_*M*__ are the mean and standard deviation of the set of *M*–snippets generated from *Z*, respectively.

### 3.3. Additional Analysis Methods

Discriminability of movement direction is assessed via two methods: the one-way variant of multivariate analysis of variance (MANOVA) and the silhouette indicator.

Simply put, MANOVA (O'Brien and Kaiser, 1985) is the generalization of the well–known analysis of variance (ANOVA) methodology. While the latter performs statistical tests regarding univariate sample means, the former compares multivariate sample means. MANOVA exploits covariance matrices to unveil correlations between dependent variables instead of the sum of squares estimator in ANOVA, which is sufficient in the univariate case. In the present work, MANOVA is utilized to determine the discriminability of movement direction based on ECoG features (either power-based or representations derived from learning on the proposed model). In particular given the four movement directions under study, MANOVA poses the null hypothesis that the multivariate means either lie on a line, on a plane or on a 3-dimensional hyperplane, being this last alternative the most discriminant option.

Silhouette values (Rousseeuw, 1987) estimate the separability of clusters of points given their labels. In the present work, average silhouette values determine the separability of movement–related representations in a 3-dimensional space. In particular for the *i*-th point, its silhouette *S*_*i*_, is defined as:

(9) $$\begin{array}{l} S_{i}=\frac{b_{i}-a_{i}}{\max\left\{a_{i},b_{i}\right\}} \end{array}$$

where *b*_*i*_ is the smallest average Euclidean distance of *i* to all points in any other cluster (where *i* is not a member), and *a*_*i*_ is the average distance between *i* and all other points belonging to the same cluster. *S*_*i*_ provides a bounded ([−1, 1]) measure of separability—average values close to −1 imply a poor clustering solution, i.e., low discrimination of features, while averages close to 1 guarantee high discriminability.

### 3.4. Parameter Selection

The proposed algorithm learns representations from ECoG ensembles in a single-channel, task-by-task basis per subject. Only the 32 channels across the frontal grid are part of the current study. To ensure a reliable baseline for the estimation of σ_*Z*_*M*__, the processing comprises the interval starting at 0.5 s before visual cue to 4 s after; yet, the subsequent statistical tests consist of timings from −0.5 to 2 s relative visual cue to emphasize TMPP samples around motor tasks (see **Figure 6**). According to previous studies related to encoding of movement-related cortical potentials (Reddy et al., 2009; Zhao et al., 2010), we focus on bursts in the high-gamma band (85–145 Hz)—a Butterworth filter with quality factor *Q* ~ 2 is utilized for this purpose. Then, *M* is set equal to 50 samples, or 100 ms, Δ = [50:10:100] ms, and γ′ = 1. The first two hyperparameters are set based on the physiology of cortical gamma rhythms and visual inspection of the traces in the time domain. The last hyperparameter is a recording-specific compromise between true and false positive detection rates in the β_*M*_ space, i.e., a value of γ = μ_*Z*_*M*__ + σ_*Z*_*M*__ guarantees a theoretical 66% of excluded *M*-snippets generated by *Z* from subsequent learning (according to an ideal Gaussian density for *Z*). All trials in the study are used for learning the prototypical high-gamma profiles. Lastly, for the present study we implement all the learning pipeline—bandpass filtering, hierarchical clustering per subject, task and channel, and feature engineering, e.g., neuromodulation rates and average timings and amplitudes—in an offline fashion.

## 4. Results

**Figure 4:**
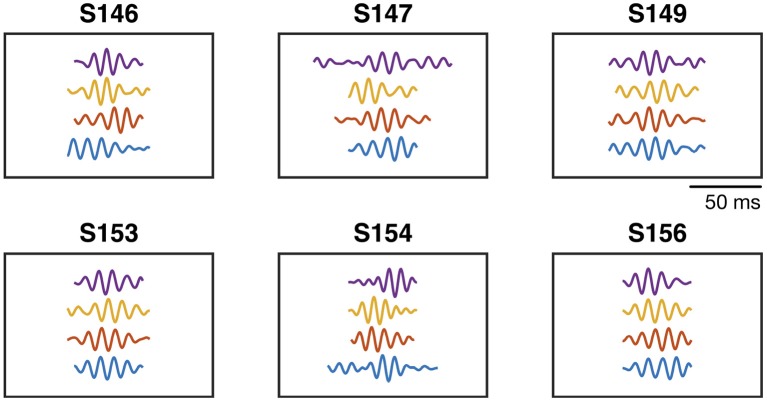
Learned sample cluster centers from high–gamma generating dictionaries. SX stands for Subject X according to the identifiers of the study. One atom per movement direction. Channel 105. All atoms have unit ℓ_2_–norm.

First, we investigate the statistics of the TMPP samples and the descriptors of the learned generating dictionaries. Table 1 emphasizes the data-driven nature of the framework: it enumerates the average number of dictionary atoms or clusters over electrodes learned by the proposed method in a subject-task-specific manner. It is worth noting that no further pruning nor post-processing of the cluster centers were performed. In terms of the learned dictionaries, Figure 4 illustrates some of the learned prototypical high-gamma micro-events for a particular channel and all subjects (one waveform per movement direction). Figure S1 highlights the variety of atoms in terms of estimated durations with respect to motor task type.

**Table 1:** Average number of learned dictionary atoms per subject and task over recording electrodes.

**Subject**	**Task**			
	**Up**	**Right**	**Down**	**Left**
146	27.15	26.28	23.59	22.87
147	32.03	32.68	28.93	27.59
149	28.87	28.00	25.28	23.84
153	32.06	34.21	36.75	36.25
154	49.90	47.34	46.00	41.87
156	29.28	30.12	31.06	30.15

*High–gamma rhythm (85–145 Hz)*.

**Figure 5:**
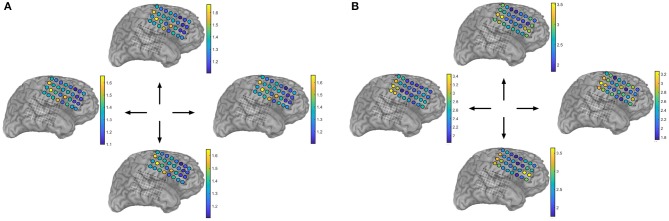
Correlation between modulated power and rate of neuromodulations in the high–gamma band (85–145 Hz). **(A)** Average high–gamma power over sensor space for each movement direction. **(B)** Average rate of high–gamma micro–events (from proposed generative model) over sensor space for each movement direction. Anterior channels (e.g., 105) display relative increase in both descriptors, yet only rate appears to be modulated depending on the movement direction. Subject 153.

**Figure 6:**
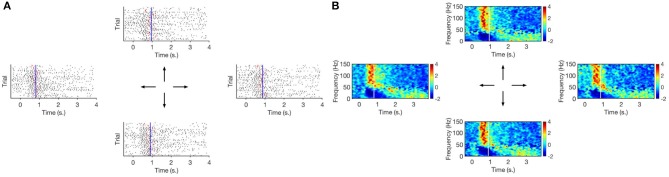
**(A)** Raster plot of high–gamma bursts (timings of TMPP, τ) per movement direction. Red ticks signal joystick movement onset for each trial. Vertical blue line is the average joystick movement onset across trials. **(B)** Corresponding spectrogram (STFT). Vertical white line is the average joystick movement onset across trials. Zero–mark indicates visual cue before movement. Motor activity lasts ~ 2.8 s. Higher rates of gamma micro–events around the 0.75 s–mark are reflected as larger densities of TMPP samples in the raster plots as well as increase of modulated high–frequency power in the spectrogram. Subject 153, channel 113.

**Figure 7:**
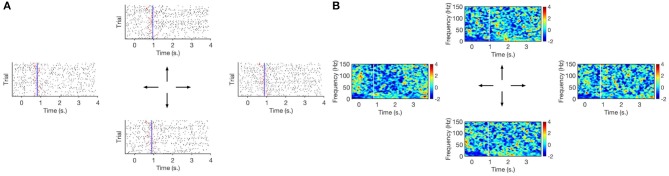
**(A)** Raster plot of high–gamma bursts (timings of TMPP, τ) per movement direction. Red ticks signal joystick movement onset for each trial. Vertical blue line is the average joystick movement onset across trials. **(B)** Corresponding spectrogram (STFT). Vertical white line is the average joystick movement onset across trials. Zero–mark indicates visual cue before movement. Motor activity lasts ~ 2.8 s. No clear indication of high–rates epochs in both raster plots and spectrograms. Subject 153, channel 101.

Next, spatial distributions are summarized; namely, Figure 5B shows the average rate of gamma bursts over channels for all movement directions. The rate statistic serves as a surrogate of the modulated power during motor tasks. This is readily confirmed in Figure 5A where average high–gamma power is illustrated instead (both features will be later used to assess and compare movement direction capabilities). Figure 6A illustrates exemplary raster plots of the timings from Subject 153, channel 113 (associated with left hand tingling according to functional mapping). An increase in *firing* of gamma events is clear around the 0.75 s—mark with respect to visual cue. Figure 6B corroborates such phenomenon by means of corresponding spectrograms (250 ms.–long segments with 50 % overlap). A clear increase in modulated high–frequency power is observed around the same 0.75 s—mark. For proper context, average joystick movement onsets are also depicted. We quantify the correlation between extracted TMPP timings and modulated gamma power by means of normalized Pearson correlation coefficients across trials, electrodes, and tasks. In particular, the correlation is performed between running sums for τ and running variances for the bandpassed traces (sliding 250 ms). Table 2 presents the means and standard deviations per subject alongside measures across patients. A similar correlation analysis (Table S2) between τ and the raw, unfiltered recordings confirm a statistically significant positive correlation between the extracted TMPP timings and the modulated high-gamma power (right-tailed two-sample *t*-test of Pearson correlation coefficients, *p* = 7.71 × 10^−263^). Lastly, the gamma firing seems to be spatially selective; for instance, channel 101 of the same subject does not display a bursting preference or clear increase in gamma power (Figure 7). This can be explained as τ being a proxy for modulated power (estimation of τ demands for power-based measures addressed in the DET).

**Table 2:** Pearson correlation coefficients between extracted TMPP timings, τ, and modulated high–gamma power (85–145 Hz) across channels, trials, and tasks.

	**Subject**						**Average**
	**146**	**147**	**149**	**153**	**154**	**156**	
Mean	0.63	0.59	0.61	0.63	0.38	0.47	0.55
Standard deviation	0.18	0.25	0.19	0.29	0.27	0.26	0.26

**Figure 8:**
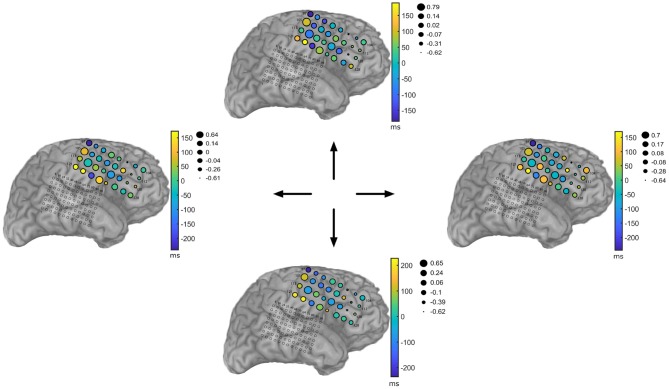
Visualization of high–gamma (85–145 Hz) Temporal Marked Point Process (TMPP) statistics over sensor space for each movement direction. Subject 153. Color scale indicates the deviation of the timings τ from the global, task–specific mean over electrodes. Radii of circles represent deviations of the weights α from the global, task–specific mean over electrodes. Log–transform of squared weight feature to encourage normality, i.e., log(α^2^).

Even though Figure 5B is informative, a more compelling picture needs to incorporate amplitude information in the form of the α feature. Figure 8 summarizes the learned TMPP timings (τ) and weight marks (α) over electrodes for each movement direction task (Subject 153). The topographical plots depict the deviations from the globals means over space, i.e., a motor task-specific spatiotemporal marked point process over the ECoG recording grid. The figures are also a succinct summary of a multidimensional array or tensor: time × amplitude × electrodes × movement direction. Similar plots for the rest of the subjects are included as Figures S2–S6.

**Figure 9:**
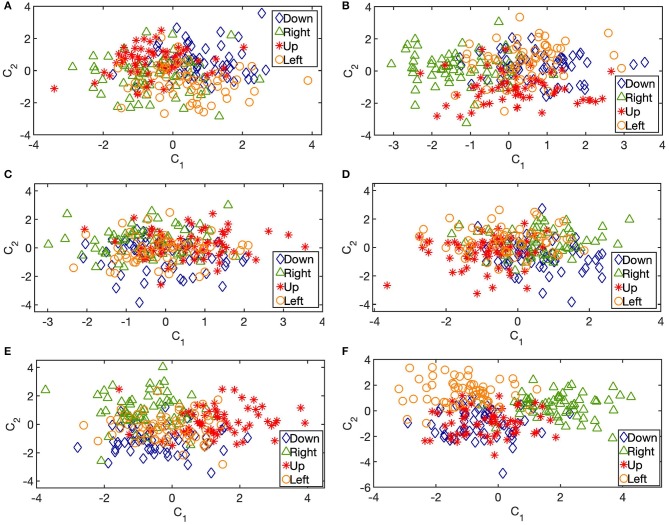
Linear projections to a 2–dimensional space that maximizes Mahalanobis distance between groups (movement directions). Byproduct of multivariate analysis of variance (MANOVA) in the interval −0.5 to 2 s relative to visual cue. **(A)** 32–dimensional log gamma power features. **(B)** 64–dimensional STFT–based power features. **(C)** 32–dimensional TMPP timing (τ) features. **(D)** 32–dimensional TMPP inter–bursts intervals (IBI) features. **(E)** 32–dimensional TMPP phasic event rate features. **(F)** 64–dimensional TMPP timing and weight (τ, α) features. Subject 153. 2–dimensional projections presented for visual purposes only. Tables 2, 3 summarize the results of similar linear projections to 3–dimensional spaces.

We begin the discriminant analysis of the learned representations in an incremental fashion. First, we focus on the timings of gamma bursts, τ. One-way MANOVA confirms that the TMPP timings are not discriminative enough for the movement direction tasks under study. Figure 9C illustrates the linear projections from the original 32-channel space to a 2-dimensional space that maximizes the separation between groups or, in this case, directions (TMPP timings from each trial are collapsed as their mean in the design matrix). Two dimensions are plotted for visual purposes. In actuality, the MANOVA results fail to reject the null hypothesis that the group means lie on a line (*p* = 0.90).

Being a point process, the micro-events might encode information in timing-dependent measures, such as inter-event intervals (or IBI—inter-bursts interval), or event rates in a similar manner as spikes in units recordings (Reich et al., 2000). Average log-IBIs constitute the labeled design matrices for the MANOVA test (log-transform to encourage normality). In particular, IBIs are calculated as the intervals (in seconds) between consecutive gamma events for a given trial. Then, the average of the logarithm of such IBIs constitute the feature for the channel/trial/task under consideration. Figure 9D shows a similar 2–dimensional linear projection that maximizes separation according to the MANOVA test. The results effectively reject the null hypothesis that the group means lie on a line (*p* = 0.0009); yet, they fail to reject the coplanar null (*p* = 0.19). Similarly, Figure 9E summarizes the linear projections corresponding to the micro-event rates, i.e., the feature for a given channel/trial/task is defined as the number of gamma bursts over the 2.5 s—interval of interest. For this case, the test rejects the null hypothesis that the means lie on a 3-dimensional hyperplane (*p* = 0.001), which is the largest possible dimension for the case of four groups. Thus, high-gamma burst rates are the most discriminative timing–related features for movement directions.

Next, we incorporate α as an additional feature. From a generative model instance, α represents the inner product between observed micro-events and closest latent dictionary atoms. We now utilize the couple {τ, α} as a 2-dimensional feature vector (TMPP timings and amplitudes from each trial are collapsed as their corresponding means in the design matrix). This novel feature can be rightfully regarded as a more refined measure of modulated power, i.e., usual TF-based feature vectors do not exploit the concept of sparse neuromodulations with localized modulated power with respect to the background and, therefore, are more likely to introduce noise to subsequent stages. Figures 9A,B confirm this limitation; the former exploits log–gamma power whereas the latter utilizes modulated gamma power over time after STFT (Short-Time Fourier Transform) decomposition (85-145 Hz for proper comparisons). On the other hand, Figure 9F shows the linear projections from the 64-bivariate ({τ, log(α^2^)}) feature space to a 2-dimensional space after the one-way MANOVA test. Classic log-gamma power features fail to reject the null hypothesis that the means lie on a 3-dimensional hyperplane (*p* = 0.20), the STFT case results in a value of *p* = 0.51, whereas a combination of timings and encoding amplitudes of the TMPP yields a rejection of said null (*p* = 3 ×1 0^−5^). Table 3 summarizes the *p*-values from similar one-way MANOVAs for all subjects across movement directions. In general, high-gamma rates and bivariate TMPP features are the most discriminant approaches while STFT power is generally more discriminant than gamma power alone. In order to normalize results across subjects, Table 4 outlines the average silhouette values for the same experiments and confirms the three most discriminant features (in descending order): bivariate TMPP features {τ, log(α^2^)}, neuromodulation rates, and Time-Frequency-based power.

**Table 3:** *p*-values from one–way MANOVA tests exploiting different types of features during the interval −0.5–2 s relative to visual cue.

**Subject**	**Feature**					
	**Power**	**STFT power**	**τ**	**IBI**	**Rate**	**τ, α**
146	8.7 × 10^−1^	8.4 × 10^−1^	9.9 × 10^−1^	**8.2 × 10**^**−3**^	**5.2 × 10^−6^**	**2.8 × 10^−2^**
147	7.3 × 10^−1^	**3.7 × 10^−2^**	6.9 × 10^−1^	9.5 × 10^−1^	**2.4 × 10^−3^**	**3.2 × 10^−2^**
149	5.8 × 10^−1^	2.1 × 10^−1^	7.6 × 10^−1^	**2.2 × 10^−2^**	**1.1 × 10^−9^**	5.6 × 10^−2^
153	2.0 × 10^−1^	5.1 × 10^−1^	9.9 × 10^−1^	4.7 × 10^−1^	**1.1 × 10^−3^**	**3.0 × 10^−5^**
154	5.5 × 10^−1^	3.0 × 10^−1^	7.8 × 10^−1^	8.3 × 10^−1^	4.9 × 10^−1^	**4.3 × 10^−3^**
156	9.6 × 10^−1^	7.1 × 10^−1^	9.2 × 10^−1^	1.7 × 10^−1^	**1.0 × 10^−5^**	**3.3 × 10^−3^**

*Rate refers to high–gamma burst rate from TMPP framework. Null hypothesis: group (movement direction) multivariate means lie on the same 3–dimensional hyperplane. p-values that lead to hypothesis rejection are marked in bold*.

**Table 4:** Average silhouette values exploiting different types of features during the interval −0.5–2 s relative to visual cue.

**Subject**	**Feature**					
	**Power**	**STFT power**	**τ**	**IBI**	**Rate**	****τ, α****
146	0.39	0.72	0.20	0.46	0.70	0.88
147	0.02	0.34	−0.004	0.16	0.48	0.44
149	0.12	0.65	0.10	0.35	0.71	0.78
153	0.03	0.14	−0.05	0.01	0.16	0.36
154	−0.03	0.03	−0.05	−0.06	−0.03	0.12
156	−0.02	0.25	−0.003	0.16	0.49	0.46
Average	0.08	0.35	0.03	0.18	0.42	0.51

*Rate refers to high–gamma burst rate from TMPP framework. Analysis on a three–dimensional space after MANOVA projections*.

Lastly, sensitivity to hyperparameters is studied. Namely, γ′ is varied in the interval [0:0.5:2] and the grand average of silhouette values are reported in Table 5. This is equivalent to modulate the sparsity of the resulting TMPP samples, i.e., smaller values of γ′ will yield dense neuromodulations over time while a higher γ′ further prunes the TMPP at expense of decreasing TPR. Yet once again, τ alone is not discriminant enough regardless of γ′. On the other hand, IBI, τ rate, and {τ, α}, show more discriminability and a slight dependency on γ′ (especially for values on the extremes of the plausible threshold interval). However, the bivariate (τ, α) features remain the most discriminant case with respect to its peers for a given noise threshold γ′.

**Table 5:** Grand average silhouette values of TMPP–based features during the interval −0.5–2 s relative to visual cue with respect to hyperparameter γ′.

**Feature**	**γ**^**′**^				
	**0.0**	**0.5**	**1.0**	**1.5**	**2.0**
τ	0.06	0.04	0.03	0.03	0.06
IBI	0.21	0.22	0.17	0.12	0.06
Rate	0.31	0.41	0.42	0.40	0.32
τ, α	0.47	0.44	0.51	0.51	0.51

## 5. Discussion

MDL principles are key in the current representation learning framework. Centroid-based clustering usually requires model selection techniques or hyperparameter tuning based on performance measures. For our case, the latter option is impractical and intractable: reconstructive cost functions such as mean-squared error between inputs and reconstructions imply the need of encoding the entire sequences in **X** ∈ R^*M*×Ψ^ when, in reality, only subsequences embedded in each sample from **X** are worth encoding. In addition, hyperparameter tuning of such a large space would be infeasible, e.g., for 10 possible number of clusters per channel, there are a total of 10^32^ = possible hyperparameter settings for the frontal ECoG grid under analysis. MDL provides a principled model selection heuristic that is able to partition the input in a hierarchical manner. Table 1 emphasizes this advantage, while at the same time, highlights the data-driven nature of the proposed algorithms. The fact that the number of dictionary atoms is different across subjects and tasks implies that diverse generative models are responsible for the inherent variability of the ECoG traces. Setting a fixed number of clusters (as is customary in centroid-based clustering) would certainly bias the learned representations. Another alternative is to compare solutions according to performance measures based on labels in a supervised fashion as in Loza et al. (2017).

### 5.1. Validation

The learning framework was initially proposed in Loza and Principe (2019) as a generalized sleep spindles detector for single-channel EEG recordings. Essentially, classic detectors either estimate the set of timings, {τ}, and a surrogate of the set of durations of the micro-events in questions (sleep spindles) or obtain amplitude, {α}, and duration features as a post-processing step (Huupponen et al., 2007; Devuyst et al., 2011; Purcell et al., 2017). Either way, both views lack the underlying generative nature the dictionary, **D**, entails.

The DREAMS database (Devuyst, 2011) was utilized to validate the methods. Single-channel (either CZ-A1 or C3-A1), 30-min-long EEG recordings from 8 subjects were made available with corresponding ground truth as visual scorings of sleep spindles from two different experts. *M* is set equal to the sample equivalent of 1.5 s while Δ is set to [0.5:0.1:1.5] s. according to scoring criteria of sleep spindles (Rechtschaffen et al., 1968; Niedermeyer and da Silva, 2005; Purcell et al., 2017). Lastly, detection performance with respect to γ′ is compared to the visual scoring annotations of expert 1. Expert 2 did not provide scorings for two subjects; therefore, it is excluded from the analysis.

Receiver operating characteristics (ROC) curves quantify the grand averages of True Positive Rates (TPR) and False Positive Rates (FPR) across subjects for a γ′ range of [−3:0.5:3] (Figure 3 in Loza and Principe, 2019). Namely, expert 1 provided ground truth as his assessment of the temporal timestamps and durations of each putative sleep spindle. On the other hand, our proposed learning algorithm returns the sets {τ, α, ω, **D**} alongside the durations of each dictionary atom or kernel in an unsupervised fashion. A true positive (TP) appears when a time sample in the EEG recording is deemed as part of a micro–event by the visual scorer and our learning algorithm simultaneously. Conversely, a false negative (FN) occurs when a time sample is deemed as part of a sleep spindle by the expert, but it is missed by the learning method. False positives and true negatives can be defined analogously. Then, TPR and FPR are calculated as:

(10) $$\begin{array}{l} TPR=\frac{TP}{TP+FN} \end{array}$$

(11) $$\begin{array}{l} FPR=\frac{FP}{FP+TN} \end{array}$$

In addition due to the inherent noisy and artifact–prone nature of EEG, the sigma index (Huupponen et al., 2000, 2007) is exploited to further reduce the FPR by filtering alpha intrusions and Electromyography (EMG) interference. Best cases of our approach correspond to a global sensitivity of 67.7% and FPR = 0.154 compared to 70.2% and 0.264 from the original report (Devuyst et al., 2011), respectively. Essentially, the proposed algorithm is able to significantly improve specificity while compromising a few TPR percentage points. At the same time, the results go beyond classic detectors by estimating generating dictionaries and features in a completely data–driven fashion. The main scope of the current manuscript is not sleep spindles detection nor optimal conditions for learning on the generative model. Yet, interested readers are referred to Loza and Principe (2019) for further information and heuristics regarding the generalized sleep spindle detector as an application of the proposed model on single-channel EEG traces.

### 5.2. Analysis of Results

Before addressing the quantitative results of our study, we devote some time to a particular set of neuromodulations that usually appear in ECoG-based epileptic studies: high-frequency oscillations (HFO) or ripples—modulated activity in the 60–100 Hz range that has been used as a biomarker to localize seizure onset zones for potential subsequent resection in medicine resistant patients (Bragin et al., 1999; Worrell et al., 2004). Even though the HFO band is a subset of the high-gamma band under study here, we believe there is no real chance of HFO leaking into our detector. Namely, as mentioned in the Experimental Setting section, all of the epilepsy patients in our study had a temporal lobe onset of epilepsy and none had a frontal neocortical onset (our 32-channel analysis takes place in the frontal grid). Also, none of the patients had a Rolandic focus of their epilepsy, which is where the recordings were taken from. Lastly, it HFO were actually leaking into the learning framework, they likely would not be synchronized to the motor tasks and would serve more as random background noise which would actually hurt, rather than strengthen our analysis.

Figure 4 underscores the data-driven solutions of the proposed methods. The learned filters are rich in terms of duration, symmetry, frequency, and modulatory patterns. This highlights the data-driven nature of the proposed framework; for instance, classic wavelets or complex sinusoids restrict the time-frequency plane to specific subsets; conversely, our learned dictionaries reflect the inherent non-stationarity of the ECoG with exceptional temporal resolution (only limited by the sampling frequency). The cluster centers depicted in Figure 4 can also be regarded as the mean values of the distributions of a mixture model that gives rise to the phasic event component in Equation (2). MDL guarantees that said clusters will not merely fit the data, but they will capture the regularity of the ECoG traces, while at the same time, keeping the model as simple as possible (simplicity is quantified here in terms of compression-based measures). Classic shift-invariant dictionary learning solutions, also deemed as convolutional sparse coding, applied to time series either require careful hyperparameter tuning or fixing the number and dimensionality of the learned atoms (Lewicki, 2002; Smith and Lewicki, 2006; Mailhé et al., 2008; La Tour et al., 2018). Our approach provides an unsupervised framework where none of those constraints are required (as previously noted, Δ is a mere blueprint for the learning algorithm to explore different dimensions, however, it is not a restrictive grid of possible phasic event durations). The price we pay, though, comes in the specialization of the EEG spectrum, i.e., all the learning is rhythm-specific (high-gamma in this case). Figure S1 summarizes the duration distributions and stands in stark contrast to traditional decomposition methods where the dictionary waveforms (e.g., complex sinusoids in Fourier analysis) have a predetermined set duration that is usually regarded as a free hyperparameter of the decomposition, e.g., window size in TF decompositions. Our proposed methods bypass this limitation by learning these duration profiles in a data-driven fashion. Further work will contemplate the potential of novel discriminative mechanisms based on the duration of gamma bursts.

Figure 5 illustrates the correlation between the high–gamma power profile and the rate of extracted micro-events over channels for each movement direction. While the power features suggest specialization over space, it does not fully indicate discriminant areas with respect to motor task type. On the other hand, the estimated rate provides a richer feature space where the neuromodulation density seems to be modulated according to movement direction. This is one of the main reasons why power-based features seem to fall short when compared to more elaborate descriptors that harness the inherent sparse nature of the phasic events (Tables 3, 4). Also, Figure 5 is a proof of concept of the proposed methods—a case in point is channel 105 where the power profile suggests an area of high local synchronization. The same channel displays high rate levels as well; however for the “up” direction, the high-gamma density slightly decreases suggesting potential discriminant behavior. Lastly, Figure 5B depicts smooth transitions in general, i.e., non-abrupt local spatial correlations that can further indicate discriminant regions (not only single electrodes) in terms of neuromodulation rates. This hypothesis is left as further work.

Figures 6A, 7A suggest specialization of gamma bursting over the cortex. Some channels increase their bursting around specific time instances, while some others do not seem to display particular distinctive patterns. This suggests a selective spectral-spatiotemporal organization of local neuronal populations in order to encode motor tasks. Similar results are observed via averaged TF decompositions, such as STFT (Figures 6B, 7B), however, the introduction of a windowing parameter blurs the temporal information encoded in the timings. Conversely, our approach provides a temporal resolution limited only by the sampling frequency: 2 ms for the current work, although the original 2034.5 Hz sampling frequency could have been used as well (yielding a ~ 0.5 ms temporal resolution with the added computational load that comes with working on higher dimensions). The MANOVA results, silhouette values and exemplary 2-D projections in Figure 9C confirm that timing information alone is not sufficient to discriminate movement directions. Yet, further work will investigate if τ might be enough to distinguish between movement and rest stages.

Figures 6, 7 also illustrate the correlation between extracted TMPP timings, τ, and modulated high-gamma power over time. Even though the recordings are aligned to the visual cue, the density of estimated gamma micro-events grows larger around the average joystick movement onset (blue lines in Figure 6). This suggests that the rate of gamma neuromodulations increases before and around movement onset on a trial-by-trial basis (see red ticks in Figure 6). The measures in Table 2 confirm the positive correlation between extracted TMPP timings and modulated high–gamma power. On the other hand, the estimated set of τ's bear no correlation (in a linear scheme) with the raw ECoG traces—average of −0.06. Comparison of these two samples (τ correlations with high-gamma filtered and raw recordings) by means of a right-tailed two-sample *t*-test confirms that the extracted phasic events follow the profile of actual high-gamma power.

A spatiotemporal marked point process succinctly summarizes the network dynamics during motor tasks. Figure 8 exemplifies a novel graphical depiction of discrete micro-events in terms of their timings and weights. Unlike TF decompositions, the topographical plots quantitatively emphasize the concept of neuromodulations and gamma bursts. For instance, electrode 105 seems to encode motor activity via large timing and weight deviations (with respect to global mean over electrodes); yet, the activity does not seem to support discrimination of movement. On the other hand, electrode 124 modulates gamma burst firing with respect to movement while keeping the amplitudes relatively the same. Most sensors seem to fall into three categories, they resemble the activity of either electrode 105 or electrode 124, or they remain relatively unaffected by the motor task, e.g., electrode 97. However, there are no regions with clear weight modulation (variability of radii across tasks). If the weight α is devised as a surrogate of power with respect to normalized bases, then the results in Tables 3, 4 and Figure 9A are completely justified—power-based measures alone that disregard timing information are not discriminant when it comes to encoding movement direction of motor tasks. This conclusion goes along the lines of Reddy et al. (2009) and Zhao et al. (2010). In the case of the two electrodes depicted in Figures 6, 7, their TMPP representations emphasize the fact that channel 101 does not actively encode motor activity—both its τ and α deviations lie close to the zeros marks, i.e., electrode 101 resembles the average global activity of the grid. On the other hand, channel 113 clearly deviates from both global trends; namely, its smaller radii suggest relatively smaller α's (again with respect to the global average of the grid for a particular task). Similarly, positive deviations from the zero-timing-mark indicate slight latencies (roughly in the order of 50 ms) with respect to the entire local neuronal population under study. This empirical analysis highlights one of the main features of the proposed algorithm: the ability to analyze EEG recordings exploiting fine temporal resolutions only limited by the sampling frequencies. Similar plots from the remaining subjects are included as Supplementary Material.

IBI and micro-event rates seem to be more suitable features to linearly separate the classes. Both features are a direct consequence of working under the premise of discrete reoccurring wave packets throughout the cortex. These representations would be infeasible for classic TF decompositions where there is no explicit notion of micro-events. While IBI estimates the average interval between gamma bursts, micro-event rates indicate the density of neuromodulations during the specified 2.5 s window. The former seems to be more discriminant than τ alone; however, the latter is consistently superior. If Figure 6 suggests a specialization in spectral-spatiotemporal organization of local assemblies, Tables 3, 4 and Figure 9E suggest a collaborative effort of the entire frontal network to modulate high-gamma burst densities at a macro level in order to sparsely encode movement direction. This conclusion could potentially lead to effective online classifiers where it would be only necessary to estimate the density of high-gamma bursts to predict motor tasks.

The incorporation of TMPP weight marks, α, into the modeling framework improves the separability of the classes and consistently outperforms all previous approaches, including classic TF-based frameworks. This last addition emphasizes the need of a generative model to encode neuromodulations as the noisy addition of weighted prototypical templates over time. STFT performs a similar generative assumption, however the basis is generic and not overcomplete; in addition, the unconstrained STFT decomposition does not encourage sparse solutions. Encoding high-gamma bursts as multimodal features not only reduces the dimensionality of the inputs, but also provides interpretable representations that can be fully validated. The bimodal representation (per channel) achieves the highest average silhouette values, signaling a proper clustering solution that can be further exploited in supervised learning frameworks, such as online BCI.

The main hyperparameter of the learning framework is the threshold γ′ of the DET. Table 5 summarizes the average silhouette values as a surrogate of the discriminability among movement directions (larger values imply better class separability). In general, τ–based results are unaffected by the choice of γ′, i.e., they all yield a poor clustering solution. When IBIs are utilized as features there is an inverse relationship between performance and γ′; this is a direct consequence of the increase in sparseness that larger γ′'s entail, i.e., temporally sparser events lead to biased IBI estimates (the same logic can be applied to τ rates). Lastly, bivariate features are not only the most discriminant solutions for a given γ′ in a consistent manner, but they also register robust intervals of the hyperparameter; consequently, this combination of features should be preferred in practice.

Now we address the concept of overfitting, i.e., merely “memorizing” the data and fitting underlying noise rather than actual trends in the ECoG. First, one of MDL's main applications is model selection (Stine, 2004; Grünwald, 2007); hence, it provides a principled framework to choose an appropriate hypothesis (or set of hypotheses) that not only explains the regularities in the data, i.e., fit it properly according to a specified criterion, but also complies with a parsimony principle that controls the complexity of such hypothesis. In this way, MDL is an explicit tool to avoid overfitting. Second, we exploit a randomization test (1,000 independent runs) that randomly shuffles the labels and proceeds to compute the MANOVA *p*-values (null hypothesis that the means lie on a 3-dimensional hyperplane) and average silhouette values for each subject. Table 6 summarizes the results and clearly indicates that no significant *p*-values emerge; silhouette-based measures are also lower than their counterparts on Table 4. In fact, the average silhouette values of Table 4 surpass the 95th percentile of the corresponding randomization test distributions in all cases except for subject 154 exploiting IBI (Table S3). In this way, we provide a proof of concept that no overfitting takes place in our study.

**Table 6:** Mean *p*-values (from MANOVA) and average silhouettes (denoted by S) after randomization test.

**Subject**	**Feature**					
	**IBI**		**Rate**		**τ**, **α**	
	***p***	**S**	***p***	**S**	***p***	**S**
146	0.83	0.17	0.83	0.17	0.66	0.73
147	0.85	0.00	0.84	0.00	0.83	0.19
149	0.84	0.11	0.84	0.11	0.77	0.54
153	0.84	−0.04	0.85	−0.03	0.84	0.07
154	0.84	−0.07	0.83	−0.05	0.84	0.00
156	0.84	0.00	0.85	0.00	0.84	0.20
Average	0.84	0.02	0.84	0.03	0.79	0.28

*Interval from −0.5 to 2 s relative to visual cue. One thousand runs of random trial shuffling were performed. γ′ = 1*.

In the previous paragraphs we glossed over an important concept for BCI deployment in real settings—online classifiers. Now, we explain in-depth how our framework can be adapted to the supervised learning setting alongside the associated theoretical and practical implications of such change. In this study, we basically clustered relevant subsequences of different lengths from single-channel, multi-trial ECoG traces. This learning is executed task by task, and the associated TMPP features and subsequent timing-related characteristics (IBI and rate) are found to be discriminant according to statistical tests and silhouette values. However, during learning, there is no cost function that maximizes discriminability (exploiting label information) and, at the same time, estimates the dictionaries and TMPP features; we only focused on the latter task. An apt analogy comes in handy here: in the computer vision field, dictionary learning is widely used; specifically, the K-SVD technique learns overcomplete, redundant dictionaries under the umbrella of sparse modeling (Aharon et al., 2006). This technique was initially utilized for compression, denoising, and demosaicking of digital images (Elad, 2010), i.e., unsupervised learning tasks similar to our framework in the present manuscript. Later, variations of K-SVD emerged in the supervised setting by exploiting label information and proposing novel cost functions (and consequently novel optimization techniques) (Zhang and Li, 2010; Jiang et al., 2013). We believe our contribution—likewise K-SVD—is the first step toward explicit discriminant models for ECoG-based BCI that exploit representation learning. To this end, the cost functions in Equations (6–8) should be modified to accommodate separability among classes (possibly via a linear classifier); then, appropriate optimization techniques (almost inevitably more complex than the algorithms presented here) would be proposed in order to estimate dictionaries that are not only adaptive, but also discriminant. If such dictionary is attainable, then online classifiers can be built on top of its atoms; for instance, a simple pipeline would assign any incoming trial to the class that minimizes the residual norm (after TMPP features estimation) according to a learned linear classifier. The computational burden and latency of said simple framework would be proportional to the added complexities of the following routines: online bandpass filtering, parallel convolutions with all of the dictionary atoms, online computation of the residue norm (per channel), and linear classifier. Clearly, more sophisticated classifiers can be built on top of such discriminant dictionary, but our goal here was to simply illustrate the point that our contribution focuses on fitting multivariate ECoG data to the proposed model (with the added model selection feature of MDL) in an unsupervised scheme, and yet, discriminability still arises as a property of the representation. In addition, this supervised learning framework would potentially allow the use of “global” dictionaries learned from a population of subjects in order to encode ECoG traces from a novel patient.

Lastly, as previously mentioned, the proposed learning algorithm is rhythm-specific. It was devised as an estimator of dictionary atoms that represent event–related oscillations at small time scales, i.e., higher frequencies. The DET exploits this constraint alongside the inherent sparsity of short-lived bursts to extract micro-events with prominent modulated envelopes. Even though the generative model of Equation (1) is general enough to explain the generative mechanisms of phasic events in the cortex, other learning frameworks are certainly needed to model non-oscillatory events (e.g., K-complexes), desynchronization type of activity (such as the decrease in beta and mu powers observed in Figure 6 prior and during joystick movement onset), and dense low-frequency events at larger time scales (e.g., phase shifts in theta and delta waves). All these cases constitute attractive new avenues of research and are left as further work. In the spirit of openness and to encourage reproducibility, the MATLAB code corresponding to the proposed methods are available at https://github.com/carlosloza/EEGMDL.

## 6. Conclusion

We proposed a generative model and learning algorithm for single-channel, multi-trial ECoG recordings that can be either posed as a convolutional variant of the sparse modeling problem where both inference and learning are attained or as an estimation of temporal marked point processes and associated prototypical activation filters. MDL is successfully exploited to render a data-driven methodology where model selection and discovery of bases from vector spaces of different dimensions are plausible. Our approach learns representations per label and models the separability among classes via optimal linear projections that maximize the Mahalanobis distance between groups. Timings and weight features of the marked point process are the most discriminative representations and outperform methodologies that do not encourage sparsity and rely on power–based measures. Further work will expand the framework to predictive modeling, i.e., jointly learning the representations as well as a classifier to effectively generalize the encoding mechanisms at work during movement direction-related motor tasks.

## Data Availability Statement

The raw data supporting the conclusions of this manuscript will be made available by the authors, without undue reservation, to any qualified researcher.

## Ethics Statement

This study was carried out in accordance with the recommendations of University of Iowa Human Subjects Review Board with written informed consent from all subjects. All subjects gave written informed consent in accordance with the Declaration of Helsinki. The protocol was approved by the University of Iowa Human Subjects Review Board.

## Author Contributions

CL was responsible for data analysis, methods, and manuscript preparation. CR was responsible for the acquisition, validation, and data sharing. SA worked on manuscript preparation and data analysis. JP collaborated with data analysis and manuscript preparation. All authors discussed the results.

### Conflict of Interest

The authors declare that the research was conducted in the absence of any commercial or financial relationships that could be construed as a potential conflict of interest.
