# Supplementary material for: Discrimination of Movement-Related Cortical Potentials Exploiting Unsupervised Learned Representations From ECoGs
Source: Front Neurosci. 2019 Nov 22;13:1248. doi: 10.3389/fnins.2019.01248 (PMC6882771; doi:10.3389/fnins.2019.01248)
Supplement: Supplementary file 1 [file Data_Sheet_1.PDF]

Supplementary Material: Discrimination of Movement–Related Cortical Potentials Exploiting Unsupervised Learned Representations from ECoGs

1 SUPPLEMENTARY TABLES AND FIGURES

1.1 Tables

Table S1. Number of trials per subject and joystick direction.

Direction	Subject					
	146	147	149	153	154	156
Down	22	42	23	56	92	40
Right	20	41	27	63	90	40
Up	20	41	20	59	90	40
Left	22	42	30	60	90	43

Table S2. Pearson correlation coefficients between extracted TMPP timings, τ, and raw, unfiltered ECoG traces across channels, trials, and tasks.

	Subject						Average
	146	147	149	153	154	156	
Mean	-0.02	-0.06	0.02	-0.24	-0.01	-0.04	-0.06
Standard Deviation	0.21	0.27	0.28	0.34	0.28	0.30	0.29

Table S3. 95–th percentile of average silhouettes distributions after randomization test. Marks indicate cases where the actual average silhouette values (Table 4) exceed the 95–th percentile of the corresponding randomization test distributions. Interval from -0.5 s to 2 s relative to visual cue. 1000 runs of random trial shuffling were performed. $\gamma' = 1$.

Subject	Feature		
	IBI	Rate	τ, α
146	0.26*	0.27*	0.81*
147	0.03*	0.04*	0.25*
149	0.19*	0.19*	0.64*
153	-0.01*	-0.01*	0.11*
154	-0.05	-0.04*	0.02*
156	0.04*	0.04*	0.26*

1.2 Figures

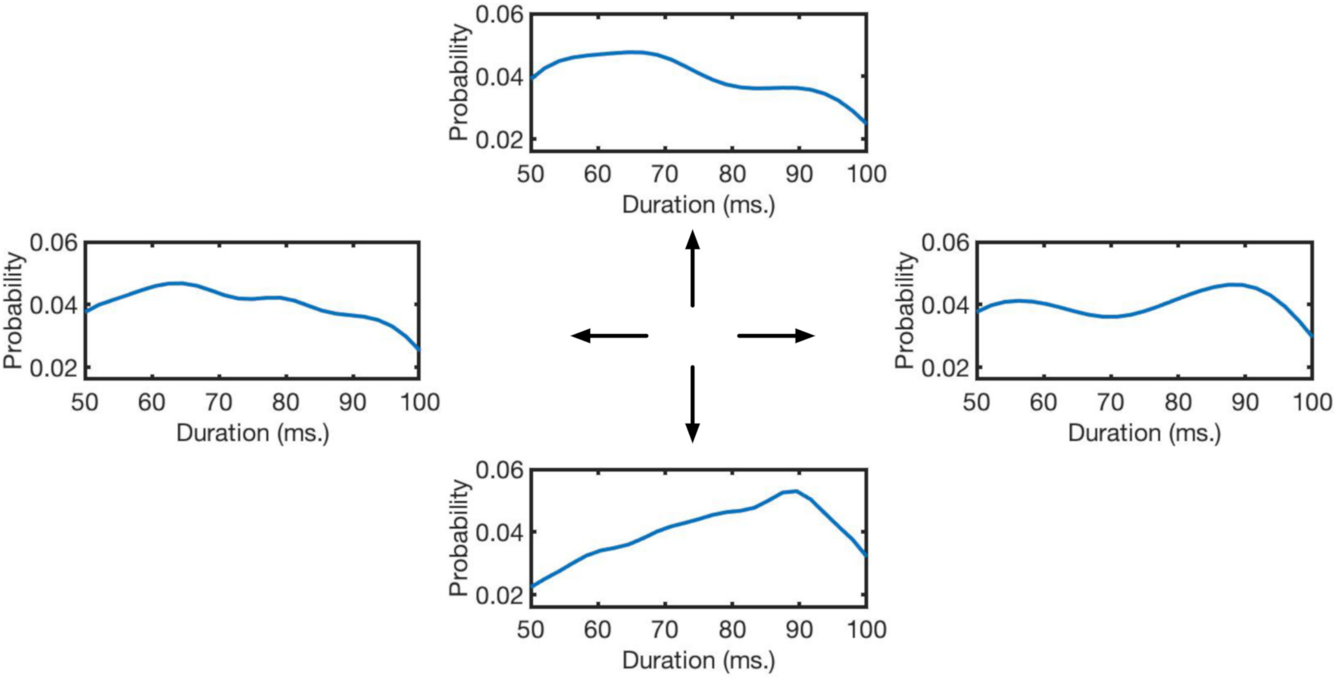

Figure S1: Estimated duration densities of learned clusters per movement direction. Subject 153, channel 113.

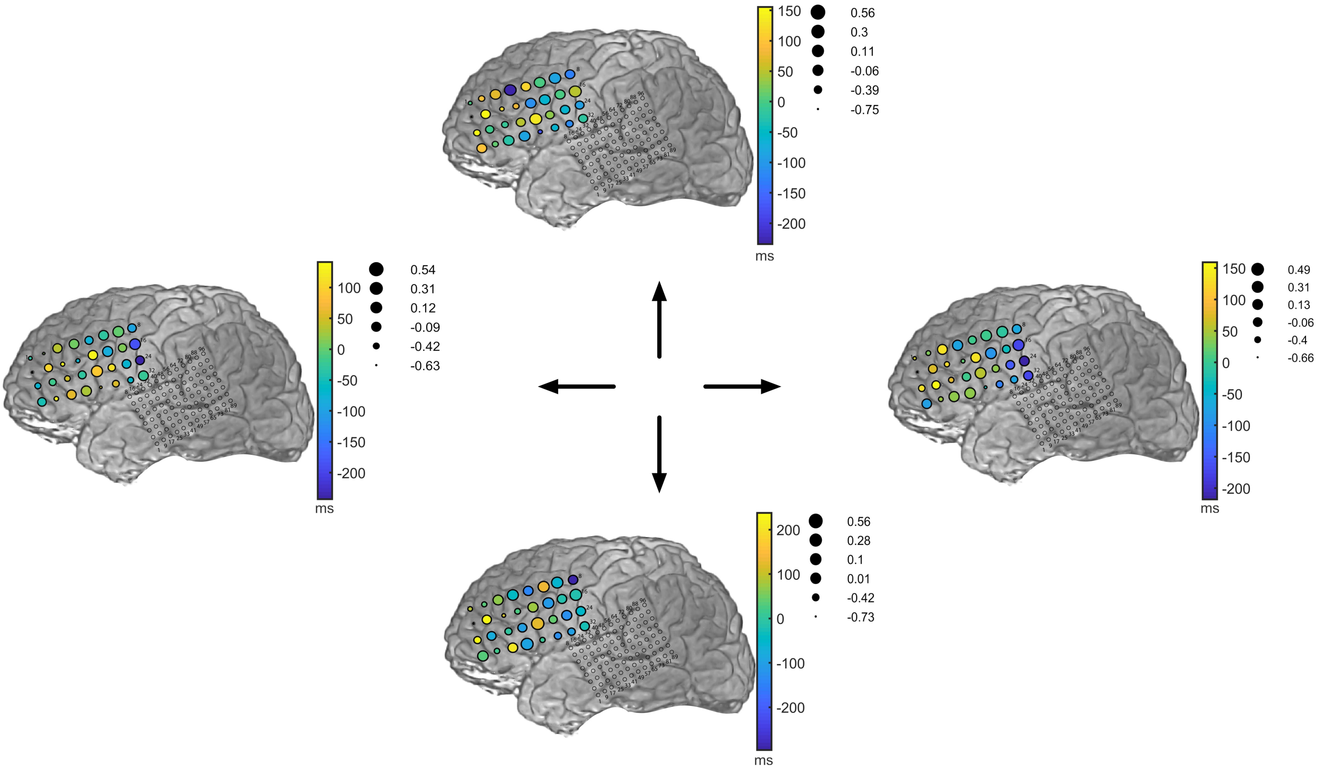

Figure S2: Visualization of high–gamma (85–145 Hz) Temporal Marked Point Process (TMPP) statistics over sensor space for each movement direction. Subject 146. Color scale indicates the deviation of the timings τ from the global, task–specific mean over electrodes. Radii of circles represent deviations of the weights α from the global, task–specific mean over electrodes. Log–transform of squared weight feature to encourage normality, i.e. $\log(\alpha^2)$

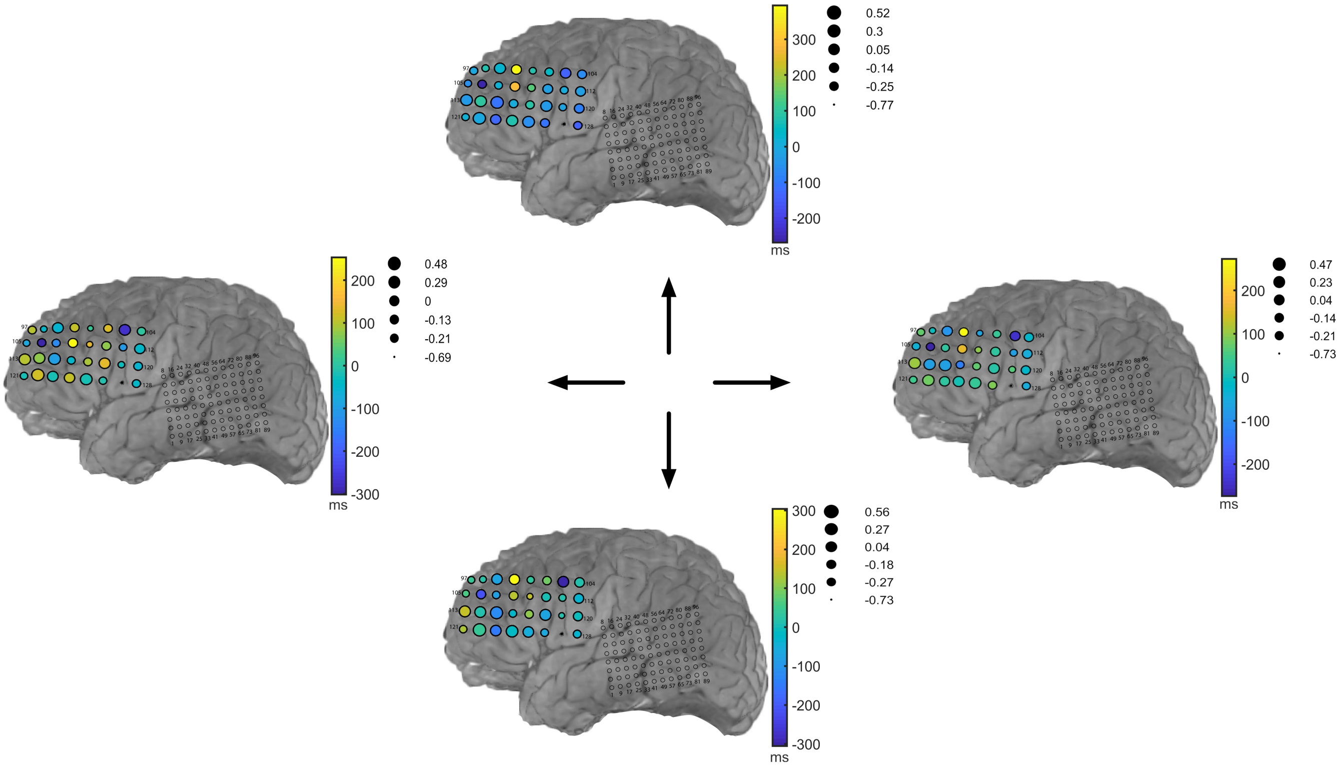

Figure S3: Visualization of high–gamma (85–145 Hz) Temporal Marked Point Process (TMPP) statistics over sensor space for each movement direction. Subject 147. Color scale indicates the deviation of the timings τ from the global, task–specific mean over electrodes. Radii of circles represent deviations of the weights α from the global, task–specific mean over electrodes. Log–transform of squared weight feature to encourage normality, i.e. $\log(\alpha^2)$

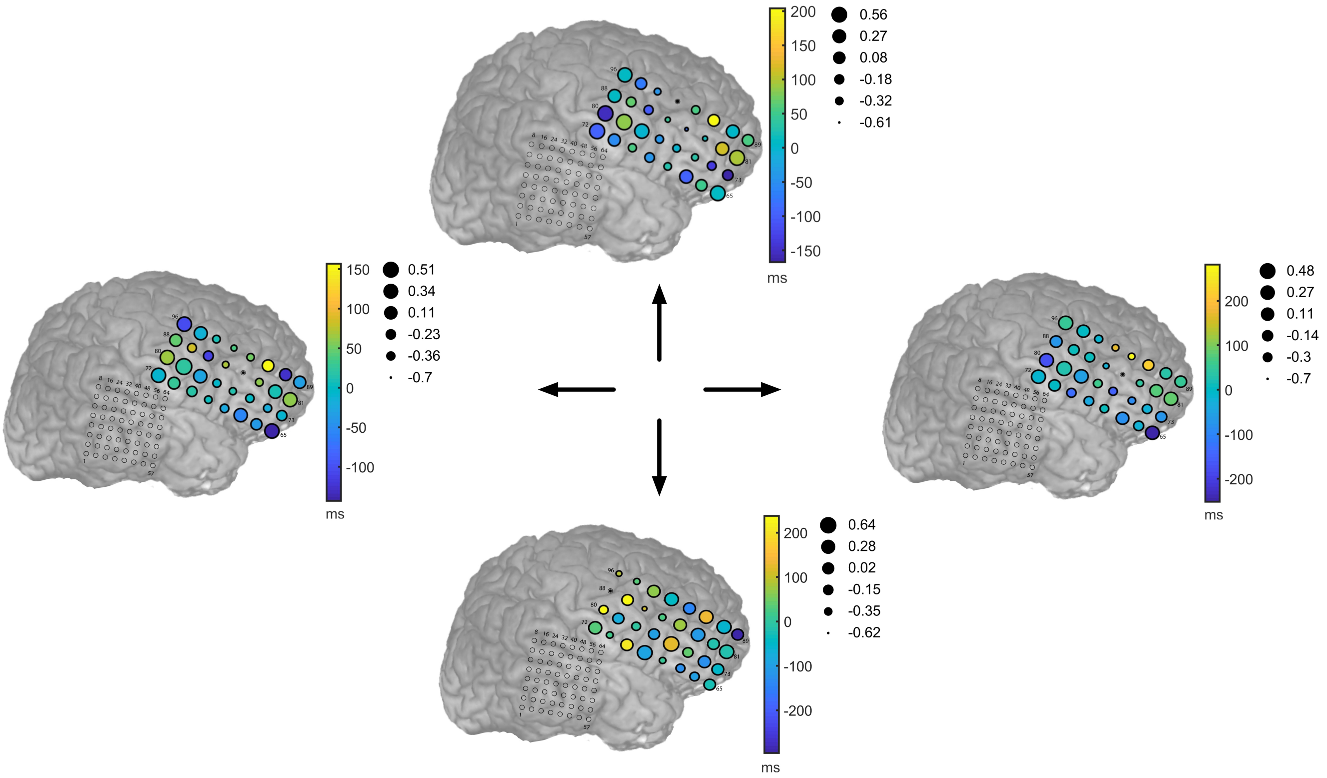

Figure S4: Visualization of high–gamma (85–145 Hz) Temporal Marked Point Process (TMPP) statistics over sensor space for each movement direction. Subject 149. Color scale indicates the deviation of the timings τ from the global, task–specific mean over electrodes. Radii of circles represent deviations of the weights α from the global, task–specific mean over electrodes. Log–transform of squared weight feature to encourage normality, i.e. $\log(\alpha^2)$

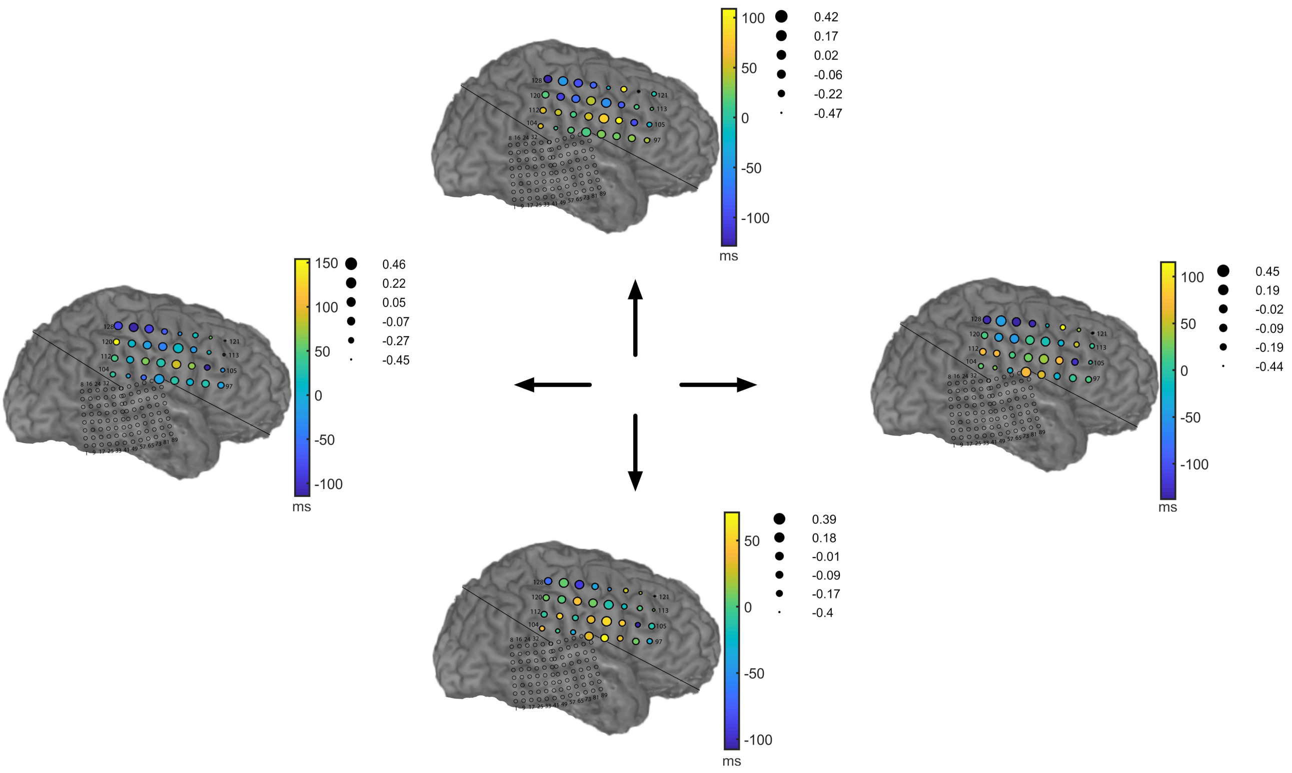

Figure S5: Visualization of high–gamma (85–145 Hz) Temporal Marked Point Process (TMPP) statistics over sensor space for each movement direction. Subject 154. Color scale indicates the deviation of the timings τ from the global, task–specific mean over electrodes. Radii of circles represent deviations of the weights α from the global, task–specific mean over electrodes. Log–transform of squared weight feature to encourage normality, i.e. $\log(\alpha^2)$

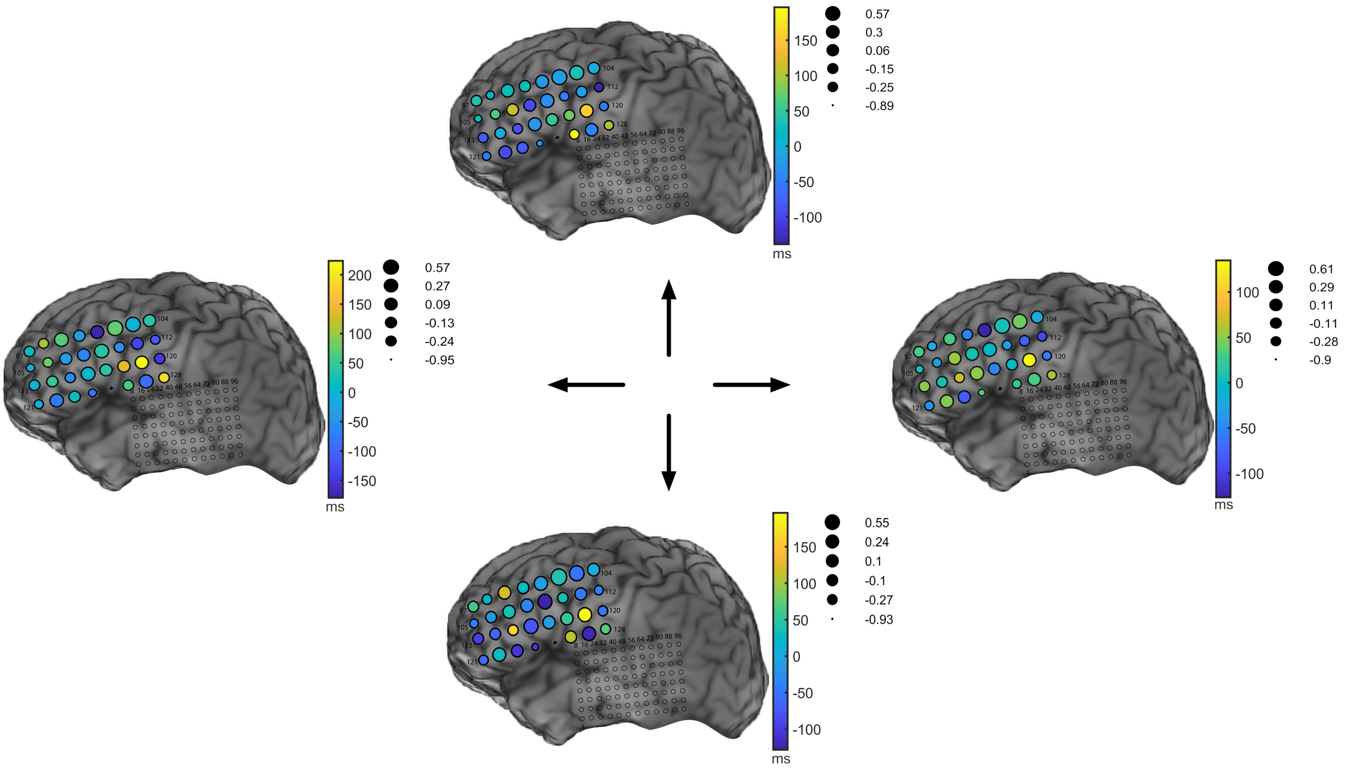

Figure S6: Visualization of high–gamma (85–145 Hz) Temporal Marked Point Process (TMPP) statistics over sensor space for each movement direction. Subject 156. Color scale indicates the deviation of the timings τ from the global, task–specific mean over electrodes. Radii of circles represent deviations of the weights α from the global, task–specific mean over electrodes. Log–transform of squared weight feature to encourage normality, i.e. $\log(\alpha^2)$
